# P04.68. Costs associated with integrative medicine interventions on chronic pain

**DOI:** 10.1186/1472-6882-12-S1-P338

**Published:** 2012-06-12

**Authors:** M Dinan, R Roberts, S Reed, R Wolever, D Abrams, R Dolor

**Affiliations:** 1 Duke Clinical Research Institute, Durham, USA; 2 Duke University Medical Center, Durham, USA; 3 UCSF Osher Center for Integrative Medicine, San Francisco, USA

## Purpose

In this study we evaluate the feasibility of using a newly formed practice-based research network of nine U.S. leading integrative medicine centers, called BraveNet, to assess the utilization and costs of integrative medicine interventions used to treat patients who suffer from chronic pain.

## Methods

Adult patients seeking their initial treatment for chronic pain at a BraveNet center were asked to participate in a prospective, observational survey. Patient-level data collection included longitudinal measures of pain, quality of life, and integrative medicine treatment utilization. Study sites provided estimates of typical charges and insurance reimbursement amounts (when appropriate) for treatments available at each center. We computed the median charge for each service as a proxy for cost and multiplied these estimates by the quantities of specific services provided to each patient.

## Results

A total of 409 patients initially enrolled in the study, with a mean duration of pain of 8 years at enrollment. A total of 322/409 (79%) patients completed a baseline visit and at least one follow-up visit at 6, 12, or 24 weeks. Over the 24 week study period these patients utilized a mean of 21.5 treatments and incurred mean costs of $2511, resulting in an average of $117 per treatment. The most frequently used therapies were acupuncture (40%) followed by massage therapy (20%), which were used an average of 11.5 and 12 times among patients receiving at least one session. Total costs varied widely by therapy, facility, and patient. Only acupuncture and integrative medicine consults were routinely reimbursed by private insurance. Mean out-of-pocket costs for acupuncture, integrative medicine consults, and massage therapy were $483, $173, and $184, respectively.

## Conclusion

Integrative medicine centers provide multiple treatment options for chronic pain. Wide variability in the cost and utilization of integrative medicine techniques poses a challenge to developing precise and generalizable estimates of cost-effectiveness.

